# Complete Genome Sequence of a Foot-and-Mouth Disease Virus of Serotype O, Isolated from Gochang, Republic of Korea, in 2016

**DOI:** 10.1128/genomeA.01671-16

**Published:** 2017-03-09

**Authors:** Taeseong Kim, Soyoon Ryoo, Jin Ju Nah, Min Geun Sagong, Sumee Lee, Kwang-Nyeong Lee, Young-Joon Ko, Jong-Hyeon Park, Myoung-Heon Lee, Sung-Hwan Wee, Dongseob Tark, Bok Kyung Ku

**Affiliations:** Foot-and-Mouth Disease Division, Animal and Plant Quarantine Agency, Gimcheon, Gyeongsangbuk-do, Republic of Korea

## Abstract

The complete genome sequence of a foot-and-mouth disease (FMD) serotype O virus isolated from Gochang, Republic of Korea, is reported here.

## GENOME ANNOUNCEMENT

Foot-and-mouth disease virus (FMDV) belongs to the genus *Aphthovirus* in the family *Picornaviridae* and causes a highly contagious vesicular disease in cloven-hoofed animal species. FMDV is divided into seven immunologically distinct serotypes, A, O, C, Asia 1, and South African Territories (SATs) 1, 2, and 3. In the Republic of Korea, serotype O has predominated since 2000 (1, 2). On 13 January 2016, FMD was diagnosed in Gochang, Jeollabuk-do, Republic of Korea, only 2 days after the first outbreak in the same province.

Here, we report the complete genome sequence of the FMDV serotype O strain (O/GC/SKR/2016) that was isolated from the vesicular fluid from an infected pig from Gochang. Viral RNA was extracted from the cell culture supernatant of the BHK-21 cell line, and cDNA was synthesized using random and oligo(dT) primers with SuperScript III reverse transcriptase (Thermo Fisher Scientific). We designed pairs of primers to produce 20 overlapping amplicons spanning the entire viral genome based on the sequence of the O/SEA/Mya-98 lineage (3). Sequence analyses were performed using SeqMan Pro version 12 (DNAStar Lasergene, USA). Phylogenetic analysis was performed using the MEGA 6.0 software.

The complete genome of strain O/GC/SKR/2016 was 8,132 nucleotides (nt) in length, including a 1,011-nt 5′ untranslated region (5′ UTR) with an 18-nt poly(C) tract and a 122-nt 3′ UTR with a ≥29-nt poly(A) tail. A single open reading frame (ORF) of 6,999 nt was predicted to encode 2,333 amino acids containing four structural and 10 nonstructural proteins. The most closely related publicly available complete genome sequence to O/GC/SKR/2016 was isolated in 2014 from Jincheon, Republic of Korea (O/SKR/JC/2014, GenBank accession no. KX162590.1), with which it shared 98.8% nucleotide and 97.9% amino acid identity. Strain O/GC/SKR/2016 shared 98.4% nucleotide and 97.7% amino acid homology with O/GJ/SKR/2016. Phylogenetic analysis of the VP1 coding sequence of the viral isolates showed that O/GC/SKR/2016 is more closely related to O/SKR/JC/2014 than O/GJ/SKR/2016 (GenBank accession no. KY086465).

### Accession number(s).

The complete genomic sequence of O/GC/SKR/2016 has been deposited in GenBank under the accession no. KY086466.
